# Safety assessment of equine allogeneic tenogenic primed mesenchymal stem cells in horses with naturally occurring tendon and ligament injuries

**DOI:** 10.3389/fvets.2024.1282697

**Published:** 2024-02-26

**Authors:** Stephanie Carlier, Eva Depuydt, Lore Van Hecke, Ann Martens, Jimmy Saunders, Jan H. Spaas

**Affiliations:** ^1^Stephanie Carlier, Kortrijk, Belgium; ^2^Department of Large Animal Surgery, Anaesthesia and Orthopaedics, Faculty of Veterinary Medicine, Ghent University, Merelbeke, Belgium; ^3^Department of Morphology, Imaging, Orthopedics, Rehabilitation and Nutrition, Faculty of Veterinary Medicine, Ghent University, Merelbeke, Belgium; ^4^Boehringer Ingelheim Veterinary Medicine Belgium, Evergem, Belgium; ^5^Boehringer Ingelheim Animal Health USA, Athens, GA, United States

**Keywords:** equine, tendonitis, desmitis, mesenchymal stem cell, primed MSCs

## Abstract

**Background:**

Mesenchymal stem cells provide a valuable treatment option in orthopedic injuries in horses.

**Objectives:**

The aim of this study was to evaluate the hematological, biochemical, immunological and immunomodulatory parameters following intralesional treatment with tenogenic primed equine allogeneic peripheral blood-derived mesenchymal stem cells (tpMSCs) in client-owned horses with naturally occurring superficial digital flexor tendon (SDFT) and suspensory ligament (SL) injuries.

**Methods:**

The immunogenicity and immunomodulatory capacities of tpMSCs were assessed in a modified mixed lymphocyte reaction, including peripheral blood mononuclear cells (PBMCs) of 14 horses with SDFT and SL injuries after treatment with tpMSCs. In a second study, 18 horses with SDFT and SL injuries received either an intralesional injection with tpMSCs (*n* = 9) or no treatment (*n* = 9).

**Results:**

The tpMSCs did not provoke a cellular immune response (*p* < 0.001) and were able to immunomodulate stimulated T lymphocytes (*p* < 0.001) *in vitro*. Therapeutic use of tpMSCs did not result in relevant hematologic or biochemical abnormalities.

**Main limitations:**

Both studies had a small sample size. No statistical analyses were performed in the second study. Fibrinogen was only analyzed in a single horse prior to treatment.

**Conclusion:**

Co-incubation of tpMSCs and PBMCs of horses that have been previously exposed to tpMSCs did not elicit a cellular immune response and tpMSCs were able to immunomodulate stimulated T lymphocytes. Intralesional treatment with tpMSCs did not provoke abnormal changes in hematological and biochemical parameters.

## 1 Introduction

Tendon and ligament lesions are one of the most common orthopedic injuries in horses (1–5). The healing of these lesions often results in the formation of scar tissue, leading to a reduction in strength and flexibility (6). This inferior tissue can have a detrimental impact on both the athletic capacity of the horse due to its’ rigorous and disorganized nature (7), but also acts as a “weak spot,” causing the tendon or ligament to be prone to re-injury (2–4, 6).

Recently, more research has been performed in order to improve the quality of tendon healing (8). The interest in orthobiologics has been especially high, with numerous studies investigating the effect of MSCs (1, 9, 10), platelet rich plasma (PRP) (11–13) and autologous protein solution (APS) (14) in a tendon model. A variety of harvesting sources of MSCs have been the focus of several papers, with bone marrow derived MSCs (BM-MSCs) reported most frequently (15–18). This current study describes the use of equine allogeneic tenogenic primed MSCs (tpMSCs) derived from peripheral blood. The benefits of using tpMSCs as an allogeneic “off-the-shelf” product is the elimination of waiting time associated with harvesting, isolation and culturing of autologous MSCs. Furthermore, the use of allogeneic donors allows for a meticulous selection process assuring consistently high quality of their MSCs. Several studies have found that the growth capacity and potency of MSCs is inversely associated with age and a declining health status (19–21). Furthermore, aging horses in active work have been shown to be more prone to tendon degeneration (22, 23), indicating the need for the use of younger, healthy horses as donors. The use of peripheral blood as a MSCs source has several advantages, namely an easy and low-invasive collection from the jugular vein, and low immunogenicity of the cells as previously shown by negative to low expression of Major Histocompatibility Complex (MHC) type I and II (24). This is in contrast to a more heterogenic expression described in adipose tissue and bone marrow-derived MSCs (25–27).

Previous studies performed by this group have shown that tpMSCs were clinically effective and safe in 66 sport horses with naturally occurring lesions of the superficial digital flexor tendon (SDFT) and suspensory ligament (SL) (28). However, clinical single and repeated use of allogeneic MSCs warrants more in depth *in vitro* and *in vivo* safety evaluation, which is investigated in the current study.

## 2 Materials and methods

The study was conducted in accordance with national and international animal welfare regulations [Directive 2001/82/EC as amended, Belgian animal welfare legislation (KB 29/05/2013), Directive 2010/63/EU and EMEA/CVMP/816/00-Final, VICH GL9 (GCP)]. Blood collection from the donor horses (EC_2018_002) was approved by the ethics committee with independent members evaluating the application as approved by the Flemish government (permit number: LA1700607). The study was performed under clinical trial authorization number 0004791 and a signed owner consent was available for each horse.

### 2.1 Investigational product and control, treatment administration, randomization and inclusion criteria

The investigational product (IVP) consisted of a proprietary formulation of equine allogeneic peripheral blood-derived tenogenic primed mesenchymal stem cells (tpMSCs) and was manufactured and qualified as previously described (10, 21, 28, 29). In summary, potential donors underwent a rigorous qualification process, including general health, infectious disease screening, and quality of stem cells. Peripheral blood was collected aseptically from the vena jugularis in sterile ethylenediaminetetraacetic acid (EDTA) tubes. Following, gradient centrifugation was performed and the interphase was isolated, seeded and cultivated in a culture medium. At passage 5, the MSCs were characterized as previously described (21) and frozen as an intermediate cell stock (ICS). Cells were then characterized, and for further differentiation ICS MSCs were thawed and cultivated. Tenogenic priming medium was added during the last passaging step for 3 consecutive days and trypsinized at 80% confluency. The MSCs were subsequently resuspended in Dulbecco’s modified Eagle medium low glucose (Life Technologies) and supplemented with 10% dimethyl sulfoxide (Sigma-Aldrich) prior to cryopreservation at −80°C, until further use. The complete manufacturing process was performed under the same high standards as for pharmaceutical products.

In the first study, two tpMSC batches isolated from two different donor horses were used. The control product (CP) consisted of 1 mL sterile saline injectable solution (0.9% NaCl). Treatment allocation was based on a randomization plan, using a ratio of 2:1 (IVP:CP) for treatment group allocation. In this study, a total of 66 horses were treated with IVP and 34 with CP (28). Fourteen horses from the IVP group were randomly selected for blood collection for peripheral blood mononuclear cells (PBMCs) isolation.

In the second study, a single tpMSC batch was used from a single donor horse. The treatment assignment was random. A total of 18 horses were included in this study, with nine horses receiving an intralesional injection with tpMSCs and nine horses receiving no intralesional treatment. Treatment allocation was ad random.

Each horse met the following inclusion criteria: a first-time unilateral tendinous or ligamentous lesion by overstrain injury, with the following findings by ultrasonography: a lesion occupying >10% of the cross- sectional area (CSA) of the tendon at the maximum injury zone (MIZ), with at least moderate hypoechogenicity (equal amount of hyper- or normo-echogenicity and hypoechogenicity) and 25–49% or less parallel fiber bundles in the lesion at the maximum injury zone. A clinical scoring was performed based on local swelling, pain to pressure and heat based on a 4-point scoring system (0 = not present, 1 = mild, 2 = moderate, 3 = severe) and lameness [American Association of Equine Practitioners (AAEP) score; 0 = not perceptible under any circumstances, 1 = difficult to observe and is not consistently apparent, regardless of circumstances, 2 = difficult to observe at a walk, or when trotting in a straight line, but consistently apparent under certain circumstances, 3 = consistently observable at a trot under all circumstances, 4 = obvious at a walk, 5 = minimal weight bearing in motion and/or at rest or a complete inability to move]. The sum of these scores had to be at least 3 in order to meet the inclusion criteria. Bilateral lesions, pre-treatment, previous participation in a stem cell study, horses with a history of lesions in the same tendinous/ligamentous structure or previous orthopedic problems that would preclude a return to full training and competition or horses with a severe medical condition that would compromise the patient’s safe participation, were excluded from the study. There was no limitation on the duration (acute, subacute or chronic) of the lesions.

Prior to intralesional injection, each horse was sedated (detomidine hydrochloride at 20 μg/kg body weight) and the region of injection was clipped and aseptically prepared. In the first study, the IVP or CP was administered intralesionally under ultrasound guidance. In the second study, the CP group did not receive an intralesional injection. To facilitate owner compliance and avoid or reduce potential local swelling caused by the needle insertion, all horses received an intravenous injection of short acting NSAIDs according to their body weight.

No ancillary therapies (e.g., shockwave, class 4 laser) or concomitant treatments (other than the single short acting NSAID-injection on day 0) were allowed during the study.

### 2.2 Safety assessments and study design

#### 2.2.1 Study 1: multicenter, blinded, placebo-controlled and randomized clinical trial (112 ± 3 days)

The first study was part of a previously reported multicenter, blinded, placebo-controlled and randomized clinical trial, with a duration of 112 ± 3 days. This study included 100 client-owned horses with a naturally occurring, first-time, unilateral lesion of the SDFT or SL (28). Cellular immunogenicity and immunomodulatory capacities of the tpMSCs were assessed in 14 of these 100 horses for the current study.

Within this study, the 14 tpMSC treated horses were randomly selected. Blood was collected through jugular venipuncture approximately 26.6 weeks after treatment (range 25.7–27.4 weeks). The blood was centrifuged, the buffy coat was collected and diluted in Hank’s balanced salt solution (HBSS, Life Technologies). Next, the suspension was layered upon an equal volume of Percoll and the interphase was collected after gradient centrifugation. Peripheral blood mononuclear cells (PBMCs) were washed and resuspended in HBSS to a concentration of 1 ×10^6^ PBMCs/mL. Subsequently, the PBMCs were labeled with carboxyfluorescein succinimidyl ester (Life Technologies), according to manufacturer’s instructions, in order to evaluate cell proliferation. Finally, PBMCs were diluted in culture medium [DMEM supplemented with fetal bovine serum, antibiotics/antimycotics and β-mercapto-ethanol (Sigma)] to a final concentration of 2 ×10^6^ cells/mL. Next, 100 μL was added to the designated wells of the u-bottom 96-well tissue-culture plate (= 2 × 10^5^ PBMCs/well). For the co-incubation samples, tpMSCs of the same donor horse and isolate were thawed, washed and resuspended in culture medium to a final concentration of 2 ×10^5^ tpMSCs/mL. The tpMSCs were plated at a 1:10 tpMSC:PMBC ratio. As a negative control, a PBMC culture from each horse was performed to assess baseline PBMC proliferation. As a positive control, PBMCs from each horse were stimulated with the mitogen concanavalin A (5 μg/mL; Sigma). Cultures were maintained for 4 days in a humidified incubator at 37°C and 95% O_2_ / 5% CO_2_. After the incubation period, PBMCs were collected, stained with 7-aminoactinomycine D (7-AAD; 1:100; BioLegend) for discrimination of live and dead cells. In the viable population, the proliferation was analyzed using a flow cytometer (BD FACSCanto II, BD Biosciences, United States). Observed proliferation would indicate a positive immune response of T lymphocytes recognizing MHC I and II molecules on MSCs surface.

#### 2.2.2 Study 2: Single center placebo-controlled study (21 days)

A single center placebo-controlled study, including 18 horses, and 21 days duration, was performed in horses suffering from naturally occurring SDFT or SL lesions.

Blood was collected on day 0 (prior to treatment in the IVP group), day 7 and day 21. The blood was analyzed for a general panel provided by the selected laboratory and included the following parameters: white blood cells (WBC), red blood cells (RBC), red cell distribution width (RDW), mean corpuscular hemoglobin (MCH), mean corpuscular hemoglobin concentration (MCHC), mean corpuscular volume (MCV), cell hemoglobin concentration mean (CHCM), Hemoglobin (Hg), hematocrit (hct), neutrophils, eosinophils, lymphocytes, monocytes, reticulocytes, reticulocyte hemoglobin content (CHr), platelet count, total protein (TP), albumin, albumin/globulin ratio, alpha-1-globulin, alpha-2-globulin, bèta-globulin, gamma-globulin, alkaline phosphatase (ALP), aspartate transaminase (AST), total bilirubin, creatine kinase (CK), creatinine, ureum, lactate dehydrogenase (LDH), gamma-glutamyl transpeptidase (GGT), glutamate dehydrogenase (GLDH), triglycerides, fibrinogen, serum amyloid A (SAA), sodium, calcium, magnesium, phosphate, potassium and iron.

Vital signs were evaluated as part of good veterinary practice on day 0, day 7 and day 21. The examining veterinarian was only obligated to report the adverse events. As such normal clinical examinations were not recorded.

### 2.3 Data analysis

For the statistical analyses of the mixed lymphocyte reaction, the normal distribution assumption was checked using the Kolmogorov–Smirnov test. Not normally distributed data were analyzed using the independent samples Mann–Whitney U Test at the 5% significance level.

Mean values and standard deviations of the clinical pathology study were calculated in Microsoft Excel^®^.

## 3 Results

### 3.1 Study 1: Multicenter, blinded, placebo-controlled and randomized clinical trial (112 ± 3 days)

**Figure 1:**
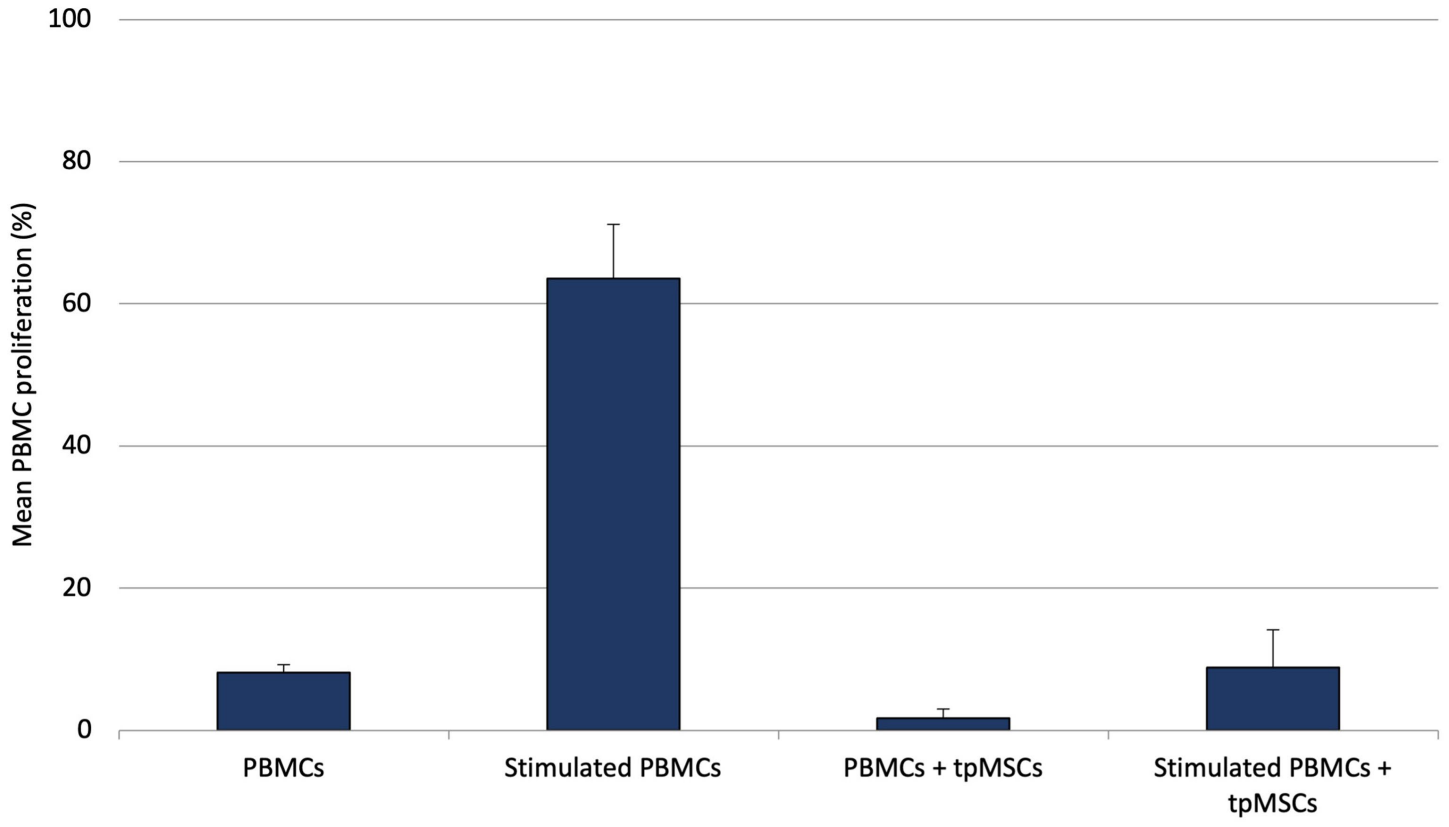
Mean PBMC proliferation (%) of equine PBMCs, stimulated PBMCs, immunogenicity (PBMCs + tpMSCs) and immunomodulation (stimulated PBMCs + tpMSCs). *p values ≤0.05.

During the *in vitro* experiment, it was found that the mean T lymphocyte proliferation percentage of the immunogenicity assay (PBMCs + tpMSCs; 1.8% ± 1.3%) was significantly lower compared to the negative control (PBMCs; 7.8% ± 1.5%; *p* < 0.001). In addition, the mean T lymphocyte proliferation percentage of the immunomodulation assay (stimulated PBMCs + tpMSCs; 8.6% ± 5.5%) was significantly lower compared to the positive control (stimulated PBMCs; 63.5% ± 7.6%; *p* < 0.001; Figure 1).

### 3.2 Study 2: single center placebo-controlled study (21 days)

Reference ranges for clinical pathology were provided by the laboratory, except for reticulocytes and CHr.

**Figure 2:**
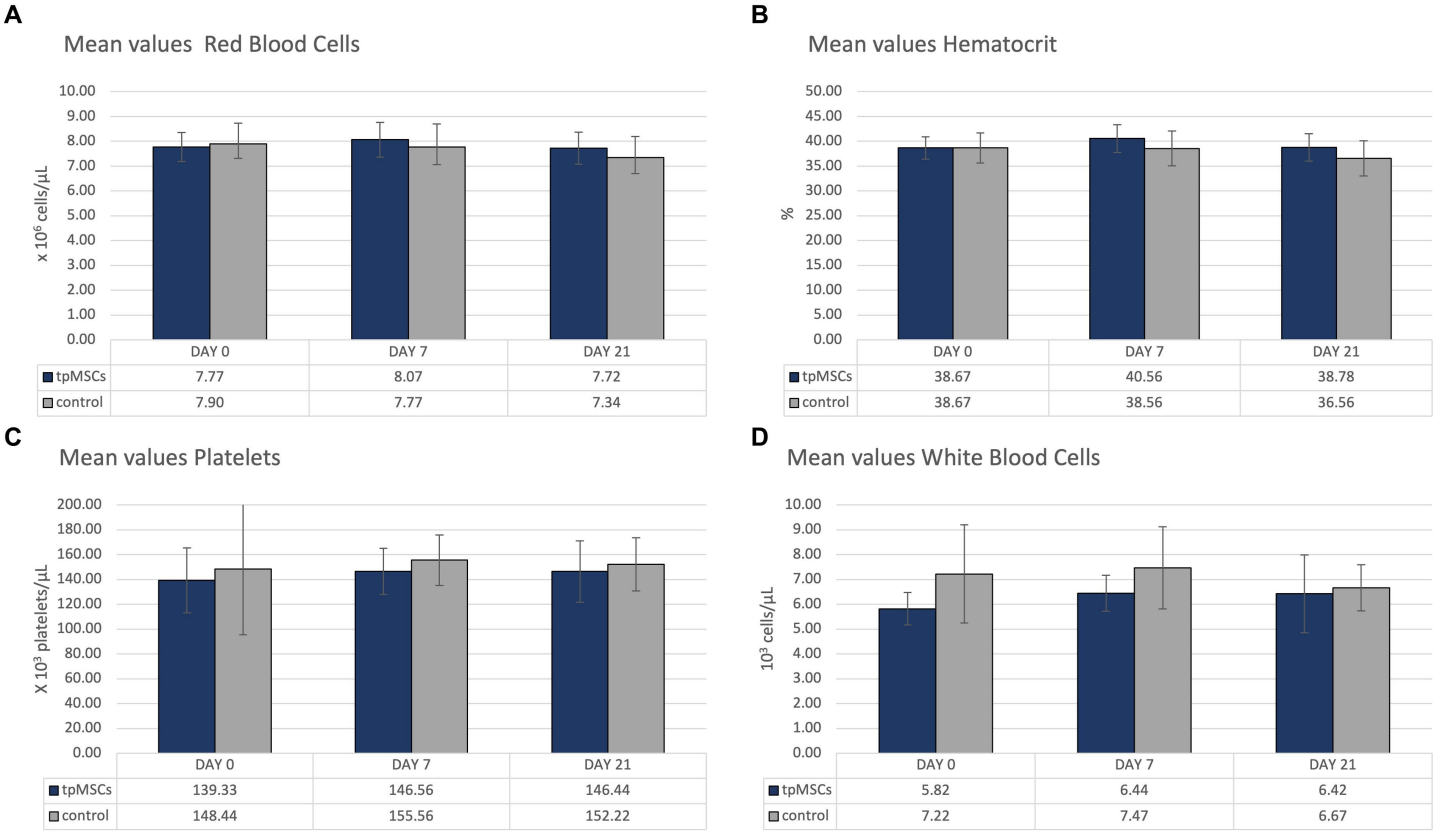
The mean values of red blood cells, hematocrit, platelets and white bloodcells.

All hematological parameters were within normal limits (Table 1; Figure 2).

**Table 1:** The table represents the mean values (standard deviation) of hematologic and biochemical parameters (except for RBC, hematocrit, platelets, WBC, fibrinogen, creatinine, total bilirubin, SAA and albumin; see Figures 2, 3).

Parameter	Treatment group	Day 0	Day 7	Day 21
Hematology				
RDW (%; 16.6–19.4%)	tpMSCs	17.21 (0.7)	16.99 (0.82)	17.01 (0.9)
	no treatment	16.8 (0.67)	16.93 (0.52)	16.99 (0.44)
Hemoglobin (g/dL) 11–16 g/dL	tpMSCs	13 (0.87)	14 (1.22)	13.33 (0.87)
	no treatment	13.44 (1.01)	13.33 (1.22)	12.67 (1.12)
MCV (fL; 42–53 fL)	tpMSCs	50.22 (2.22)	50.44 (2.01)	50.33 (2.5)
	no treatment	49.33 (2.29)	49.89 (2.42)	50 (2.00)
MCH (pg; 14–18 pg)	tpMSCs	16.89 (0.60)	17 (0.71)	17.11 (0.78)
	no treatment	16.78 (0.83)	17.33 (0.87)	17.22 (0.83)
MCHC (g/dL; 33–36 g/dL)	tpMSCs	34 (0.71)	34.11 (0.60)	34.11 (0.78)
	no treatment	34.33 (0.71)	34.11 (0.60)	34.44 (0.53)
CHCM (g/dL; 33–36 g/dL)	tpMSCs	33.46 (0.81)	33.29 (0.72)	33.34 (0.89)
	no treatment	33.62 (1.18)	33.31 (0.68)	33.27 (0.61)
Neutrophils segments (%; 45–70%)	tpMSCs	55 (9.37)	56.44 (5.61)	55.33 (6.54)
	no treatment	58.67 (7.58)	59.67 (4.64)	56.56 (8.14)
Lymphocytes (%; 20–45%)	tpMSCs	37.56 (9.91)	35.78 (5.12)	36.22 (5.19)
	no treatment	36.44 (8.28)	34 (4.56)	36.67 (8.15)
Monocytes (%; 2–6%)	tpMSCs	4.00 (0.71)	4.33 (1.12)	4.44 (1.42)
	no treatment	3.11 (1.36)	3.89 (0.78)	4.00 (1.00)
Eosinophils (%; <4%)	tpMSCs	2.44 (0.88)	2.44 (1.24)	2.89 (3.14)
	no treatment	0.89 (0.6)	1.44 (0.53)	1.56 (0.88)
Basophils (%; <2%)	tpMSCs	0.67 (0.5)	0.44 (0.53)	0.67 (0.71)
	no treatment	0.44 (0.53)	0.56 (0.53)	0.67 (0.71)
Neutrophil segments (×10^3^/μL; 2.5–5.9 ×10^3^/μL)	tpMSCs	3.19 (0.49)	3.62 (0.33)	3.49 (0.68)
	no treatment	4.36 (1.85)	4.47 (1.13)	3.71 (0.40)
Lymphocytes (×10^3^/μL; 1.4–3.9 ×10^3^/μL)	tpMSCs	2.22 (0.69)	2.32 (0.53)	2.32 (0.68)
	no treatment	2.51 (0.44)	2.52 (0.62)	2.49 (0.86)
Monocytes (×10^3^/μL; 0.1–0.5 ×10^3^/μL)	tpMSCs	0.24 (0.05)	0.27 (0.09)	0.29 (0.09)
	no treatment	0.20 (0.10)	0.28 (0.07)	0.27 (0.09)
Eosinophils (×10^3^/μL; 0–1 ×10^3^/μL)	tpMSCs	0.14 (0.05)	0.17 (0.07)	0.23 (0.33)
	no treatment	0.08 (0.04)	0.11 (0.03)	0.12 (0.04)
Basophils (×10^3^/μL; <0.2 ×10^3^/μL)	tpMSCs	0.07 (0.05)	0.04 (0.05)	0.06 (0.07)
	no treatment	0.04 (0.05)	0.06 (0.05)	0.06 (0.05)
Reticulocytes [/1,000 erythrocytes; 0.18–1.35 reticulocytes/1000 erythrocytes (30)]	tpMSCs	0.59 (0.19)	0.77 (0.29)	0.68 (0.20)
	no treatment	0.67 (0.17)	0.68 (0.14)	0.79 (0.26)
Reticulocyte hemoglobin content [pg; 14–23 pg. (31)]	tpMSCs	18.47 (0.74)	18.46 (1.36)	18.09 (0.75)
	no treatment	18.09 (0.98)	18.07 (0.88)	17.5 (0.85)
Biochemistry				
Iron (μg/dL; 89–227 μg/dL)	tpMSCs	174.67 (58.8)	165.67 (47.99)	169.89 (39.44)
	no treatment	164.78 (54.32)	149.67 (61.32)	171.78 (47.42)
Sodium (mEq/L; 135–144 mEq/L)	tpMSCs	140 (2.60)	140.78 (2.77)	138.11 (2.03)
	no treatment	139.78 (2.49)	139 (2.69)	139.33 (1.41)
Potassium (mEq/L; 3.2–5.1 mEq/L)	tpMSCs	4.07 (0.56)	4.19 (0.73)	4.42 (0.4)
	no treatment	4.21 (0.83)	4.2 (1.03)	4.08 (0.97)
Calcium (mg/dL; 11.7–13.6 mg/dL)	tpMSCs	12.68 (0.36)	12.66 (0.48)	12.48 (0.44)
	no treatment	12.07 (0.34)	12.31 (0.62)	12.36 (0.49)
Phosphate (mg/dL; 2.2–4.1 mg/dL)	tpMSCs	2.68 (0.42)	2.93 (0.3)	2.86 (0.49)
	no treatment	3.31 (1.13)	2.6 (0.76)	3 (0.51)
Magnesium (mg/dL; 1.3–2.3 mg/dL)	tpMSCs	1.93 (0.21)	1.97 (0.15)	2.01 (0.18)
	no treatment	1.98 (0.2)	2.02 (0.19)	1.98 (0.13)
Ureum (mg/dL; 20–41 mg/dL)	tpMSCs	27 (7)	30.67 (6.56)	25.11 (6.66)
	no treatment	24.44 (5)	25.67 (6.65)	23 (4.69)
CK (IU/L; 0–500 IU/L)	tpMSCs	229.56 (80.63)	237.56 (35.89)	240.56 (66.07)
	no treatment	340.33 (396.22)	190 (61.04)	269 (92.36)
AST (IU/L; <385 IU/L)	tpMSCs	279.89 (41.04)	295.33 (30.27)	285.67 (34.45)
	no treatment	291.33 (38.53)	280.56 (43.43)	271 (26.31)
LDH (IU/L; <600 IU/L)	tpMSCs	336.44 (72.8)	363.56 (49.97)	381.33 (74.94)
	no treatment	368.33 (138.81)	305.89 (84.81)	366.11 (100.12)
GGT (IU/L; 0–30 IU/L)	tpMSCs	13.44 (4.3)	15.44 (4.1)	16.22 (4.87)
	no treatment	21.44 (13.04)	18.33 (8.73)	16.67 (5.83)
ALP (IU/L; <250 IU/L)	tpMSCs	128.56 (32.21)	149.33 (37.25)	147.56 (44.62)
	no treatment	146.78 (39.03)	140.22 (33.7)	132.78 (24.02)
GLDH (IU/L; 0–10 IU/L)	tpMSCs	3.89 (2.71)	4.00 (5.41)	4.11 (1.96)
	no treatment	2.56 (1.88)	2.67 (0.87)	4.00 (1.58)
Triglycerides (mg/dL; >50 mg/dL)	tpMSCs	26 (7.68)	24.67 (7.6)	28.56 (7.09)
	no treatment	24.22 (8.71)	31.33 (3.97)	30.56 (8.8)
Total protein (g/dL; 5.5–7.5 g/dL)	tpMSCs	6.09 (0.37)	6.42 (0.32)	6.29 (0.47)
	no treatment	6.34 (0.35)	6.28 (0.41)	6.09 (0.43)
Albumin (%; 39–53%)	tpMSCs	52.9 (2.11)	51.83 (4.66)	51.48 (5.07)
	no treatment	49.16 (3.26)	48.09 (3.74)	48.8 (3.46)
Alpha-1 globulin (%; 2–6%)	tpMSCs	4.47 (1.08)	4.51 (1.19)	4.41 (1.37)
	no treatment	4.29 (1.27)	4.66 (1.31)	4.59 (1.21)
Alpha-1 globulin (g/dL; 0.1–0.4 g/dL)	tpMSCs	0.27 (0.08)	0.3 (0.09)	0.29 (0.11)
	no treatment	0.27 (0.07)	0.29 (0.08)	0.28 (0.08)
Alpha-2 globulin (%; 10–16%)	tpMSCs	11.46 (1.1)	11.59 (1.26)	11.30 (1.25)
	no treatment	11.88 (1.36)	12.09 (1.76)	11.62 (1.58)
Alpha-2 globulin (g/dL; 0.6–1 g/dL)	tpMSCs	0.69 (0.09)	0.74 (0.09)	0.71 (0.12)
	no treatment	0.76 (0.09)	0.77 (0.1)	0.72 (0.1)
Beta globulin (%; 12–23%)	tpMSCs	16.66 (2.26)	16.51 (3.08)	15.82 (3.45)
	no treatment	18.14 (1.45)	18.32 (2.08)	16.01 (3.68)
Beta globulin (g/dL; 0.6–1.5 g/dL)	tpMSCs	1.01 (0.18)	1.06 (0.19)	0.99 (0.26)
	no treatment	1.17 (0.14)	1.14 (0.17)	0.97 (0.19)
Gamma globulin (%; 0–22%)	tpMSCs	14.51 (3.08)	15.56 (3.81)	16.99 (4.47)
	no treatment	16.56 (2.28)	16.84 (2.11)	18.98 (5.04)
Gamma globulin (g/dL; 0.6–1.5 g/dL)	tpMSCs	0.89 (0.2)	1.00 (0.27)	1.07 (0.33)
	no treatment	1.06 (0.17)	1.08 (0.16)	1.17 (0.4)
Albumin/Globulin ratio (0.6–1.1)	tpMSCs	1.13 (0.11)	1.10 (0.17)	1.08 (0.2)
	no treatment	0.98 (0.14)	0.94 (0.17)	0.97 (0.14)

Mean values outside of the provided reference range are marked in bold. Fibrinogen analysis was only performed in a single horse on day 0*.

**Figure 3:**
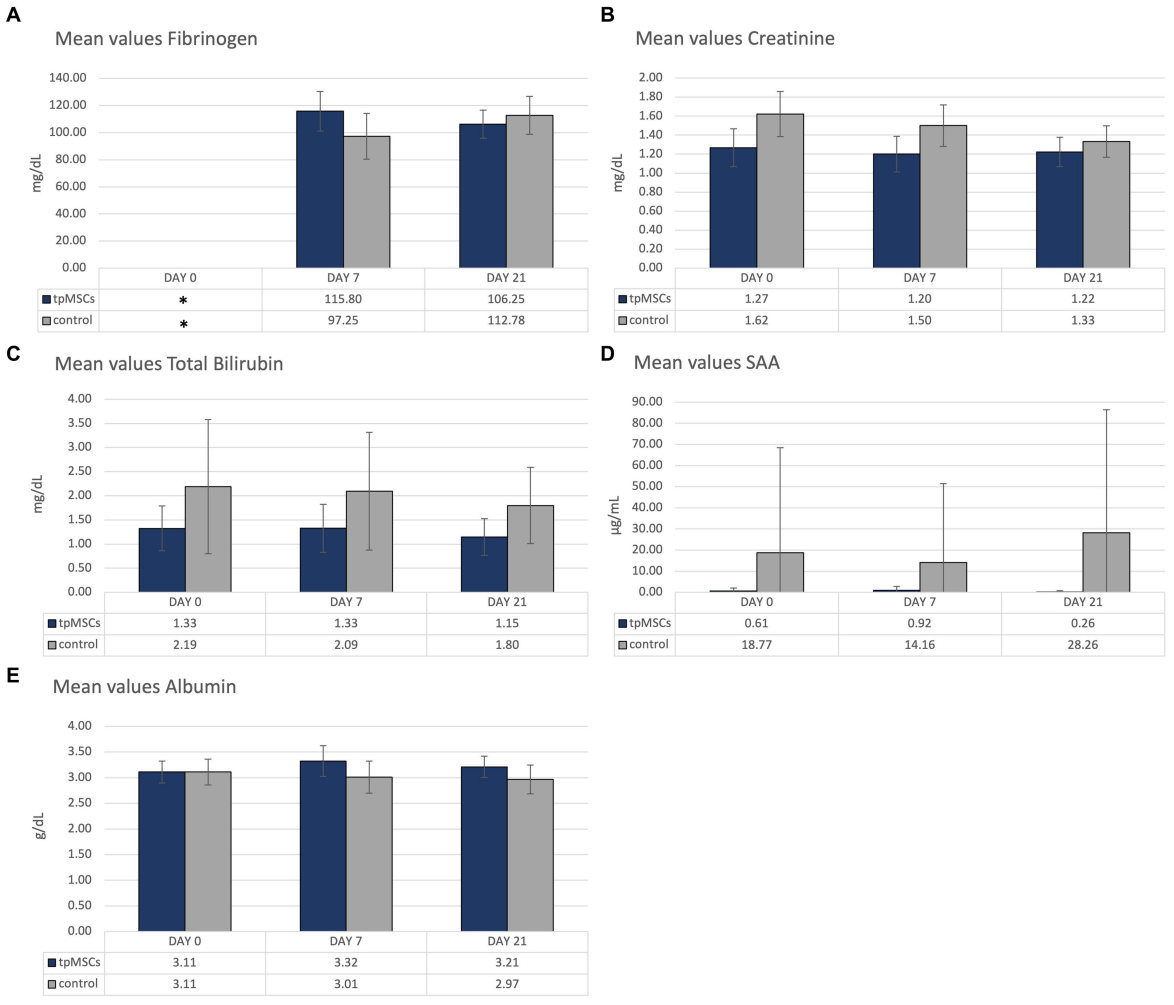
Biochemistry: mean values of fibrinogen (A), creatinine (B), total bilirubin (C), SAA (D) and albumin (E) of the horses treated with tpMSCs and horses that received no treatment (control). *On day 0, fibrinogen was only analyzed in a single horse.

Biochemistry parameters that were outside of the reference range are depicted in Figure 3. All other mean values were within the provided reference range (Table 1). No abnormalities were found in clinical exams in any of the horses during the study.

Due to a human error, fibrinogen on day 0 was only analyzed in one horse, in the IVP group. Therefore, day 0 fibrinogen is excluded from the figure (Figure 3A). In both groups, values outside of the reference range of fibrinogen (100–200 mg/dL) occurred. As previously mentioned, day 0 fibrinogen was analyzed in a single horse, from the IVP group, and had a value of 72 mg/dL. In the CP group, the mean value of fibrinogen on day 7 was 97.25 ± 16.96 mg/dL (Figure 3A). The mean value of creatinine was slightly above the reference range (1.62 ± 0.24 mg/dL; reference range: 0.8–1.5 mg/dL; Figure 3B). The mean total bilirubin in the control group was outside of the reference range (0.5–2.0 mg/dL) on day 0 and 7 (2.19 ± 1.39 mg/dL and 2.09 ± 1.22 mg/dL, respectively; Figure 3C). The mean SAA in the control group was increased on all timepoints (reference <4 μg/mL; 18.77 ± 49.74 μg/mL, 14.16 ± 37.31 μg/mL and 28.26 + 58.14 μg/mL on day 0, 7 and 21, respectively; Figure 3D). Finally, the mean albumin concentration was marginally increased on day 7 in the IVP group (3.32 ± 0.30 g/dL; reference range: 2.4–3.3 g/dL; Figure 3E). All other mean values were within the provided reference range (Table 1). All horses were in good clinical health.

## 4 Discussion

This paper discusses the immunogenic and immunomodulatory capacities of tpMSCs *in vitro* and investigates their safety through hematological and biochemical parameters in treated horses with naturally occurring tendon and ligament injuries.

To support the hypothesis that tpMSCs are devoid of MHC type II and low in type I and thus evade immune rejection, an *in vitro* assay was performed evaluating the response of PBMCs in the presence of tpMSCs. MHC type I and II-mediated responses primarily include activation of T lymphocytes, including memory T cells (32), making them more sensitive to subsequent exposure of the antigen. In order to evaluate increased sensitivity of PBMCs due to previous exposure to tpMSCs, PBMCs of horses that had already been treated with tpMSCs were used for co-incubation with tpMSCs from the same stem cell isolate (and therefore the same donor horse) that was used for treatment. Co-incubation of PMBCs and tpMSCs did not provoke a cellular immune response. Moreover, tpMSCs were able to reduce the proliferation of stimulated PBMCs in an *in vitro* inflammatory response, which suggests that MSCs have a dampening effect on inflammation. This corresponds to the finding that inactivation of MHC type I and II leads to hypo-immunity in pluripotent stem cells (33) and to the described immunomodulatory capacities of MSCs (34–36). It has been described that the treatment of MScs with growth factors can potentially influence the MHC expression (37, 38). However, the hypo-immunity of the tpMSCs in an *in vitro* environment is confirmed *in vivo* by the lack of treatment related adverse events following the intralesional injection of tpMSCs in equine tendon lesions, in both natural and experimental settings (10, 28, 39).

Study one and 2 differed in CP design, where intralesional saline was injected in study 1, and no treatment was opted for by attending clinicians in study 2. In the first study this was imperative as a control, as the injection of a certain volume could potentially lead to aggravation of the lesion (40).

The aim of the single center placebo-controlled clinical pathology study was to evaluate the safety of tpMSCs by assessing the occurrence of possible abnormal changes in hematological and biochemical parameters following tpMSC-treatment. During the study, no adverse events were reported, and all mean parameters of the IVP-group remained within the reference ranges following treatment, with the exception of the mean albumin on day 7. On day 7, the mean (absolute) albumin level was marginally increased (3.32 g/dL; reference range: 2.4–3.3 g/dL) in the IVP group. Albumin ranges in healthy horses have been reported to be up to 4.2 g/dL (41). Moreover, the mean relative values for albumin were within the reference range. Hyperalbuminemia is most commonly associated with dehydration (42, 43). However, there was no indication that the horses in the IVP group were dehydrated based on the other hematological and biochemical parameters. Furthermore, the relevance is questionable as the value can be considered normal, based on published reference values.

By mistake, fibrinogen was only analyzed in a single horse on day 0. This horse belonged to the IVP-group and had a value below the reference range (72 mg/dL; reference range: 100–200 mg/dL). Hypofibrinogenemia can indicate liver failure and disseminated intravascular coagulation (44). However, the horse was in good general health and there was no other indication (nor in clinical presentation nor in clinical pathology) or liver disease or DIC. The fibrinogen level of the horse was lower than normal prior to treatment and had normalized by the following timepoint. Furthermore, the mean fibrinogen of the IVP-group remained within the reference range following treatment. In conclusion, the mean fibrinogen in the IVP-group was only out of reference prior to treatment and is therefore unrelated to the treatment. In the control group there was a marginally lower mean fibrinogen (97,25 mg/dL) on day 7.

The mean values of creatinine, total bilirubin and SAA were out of range on one or more timepoints in the control group. As none of the horses in the control group received the IVP-treatment, these outliers are considered unrelated to the tpMSCs treatment.

It should be noted that this study included a small sample size and that no statistical analyses was performed. To assess significant changes or trends compared to baseline values and a control group, further studies with larger sample sizes are warranted. Regardless, the purpose of this study was to identify the possible occurrence of abnormal changes in hematological or biochemical parameters in order to assess the safety of the intralesional use of tpMSCs in naturally occurring tendinitis or desmitis. As such we can conclude that only the mean of a single parameter was marginally increased following tpMSC treatment and can be considered normal based on published reference ranges, whereas multiple parameters were out of range in the control group.

A single dose of NSAID was administered for animal welfare reasons, to avoid unnecessary pain and swelling due to the needle insertion all animals treated with intralesional injection (IVP and CP). The use of NSAIDs could potentially have a negative impact on the potency of MSCs (45, 46), however It is unclear whether the concomitant use of NSAIDs interferes with the working mechanism of the tpMSCs. In the previously performed clinical field trial efficacy was demonstrated for tpMSC in naturally occurring tendon and ligament injuries, with statistically significant differences in all clinical and ultrasonographical parameters between the groups, as well as by significantly lower reinjury rate in tmMSCs treated horses in the long term (28). This data suggests that NSAIDs did not impact the potency of the tpMSCs. An additional blinded study should be performed to confirm this hypothesis.

## 5 Conclusion

TpMSCs did not elicit a cellular response of T lymphocytes in the previously treated horses, and were able to immunomodulate stimulated T lymphocytes in an *in vitro* environment. Previous *in vivo* exposure to tpMSCs did not appear to affect the T lymphocytes in subsequent co-incubation with tpMSCs. Furthermore, the use of tpMSCs did not cause hematological or biochemical abnormalities. Based on these findings and previously reported clinical safety assessments, tpMSCs can be considered a safe treatment for tendon and ligament lesions in horses.

## Data availability statement

The raw data supporting the conclusions of this article will be made available by the authors, without undue reservation.

## Ethics statement

The animal studies were approved by LA1700607 (Ethics committee Boehringer Ingelheim Veterinary Medicine Belgium). The studies were conducted in accordance with the local legislation and institutional requirements. Written informed consent was obtained from the owners for the participation of their animals in this study.

## Author contributions

SC: Investigation, Writing – original draft. ED: Conceptualization, Investigation, Writing – review & editing, LV: Writing – review & editing. AM: Writing – review & editing. JS: Writing – review & editing. JHS: Conceptualization, Writing – review & editing.
